# Effects of person-environment fit of gender-role orientation on burnout, engagement and hair steroids as stress biomarkers among women

**DOI:** 10.1186/s12995-021-00303-5

**Published:** 2021-04-16

**Authors:** Eva Wacker, Axel Fischer, Julia Schorlemmer

**Affiliations:** 1 FOM Hochschule für Oekonomie und Management Berlin, Institut für Gesundheit und Soziales, Bismarckstr. 107, 10625 Berlin, Germany; 2 Charité – Universitätsmedizin Berlin, Campus Virchow-Klinikum, Augustenburger Platz 1, 13353 Berlin, Germany

**Keywords:** Person-environment fit, Gender-role orientation, Burnout, Engagement, Hair steroids

## Abstract

**Background:**

Analysis on gender related differences in occupational stress and burnout levels usually reveal higher occupational stress and burnout levels for women compared to men, especially in male-dominated working environments. In opposition to group differentiation, more specific gender-related dimensions feminity and masculinity were used in the study to describe individual and work environment characteristics and analyze their effects.

**Methods:**

In a cross-sectional design, survey results were linked to steroid levels in hair samples. Data was collected in a German medical services company with 146 employed women age 22–66 years (*M* = 40.48, *SD* = 10.38), 58 of them provided hair samples for steroid detection. Feminity and masculinity were measured by Gender Role Orientation Scale GTS+. Two Person-Environment fit scores in feminity and masculinity were calculated by subtracting individual from environment values. Both fit scores were proved as predictors in hierarchical linear regression models predicting burnout and work engagement as well as hair steroids cortisol, cortisone, DHEA, testosterone and progesterone detected by Liquid Chromatography-Mass Spectrometry (LC-MS/MS) as stress biomarkers. Bivariate correlations as well as moderator and mediator analysis were implemented.

**Results:**

After considering age, role clarity, and work organization, Person-Environment fit in feminity still added significant variance explanation (β = .23, *∆ R*^*2*^ = .05, *p* = .003) for burnout. Person-Environment fit in feminity also explained poor variance in work engagement (β = −.29, *R*^*2*^ = .09, *p <* .001). Person-Environment fit in masculinity added considerable variance explanation (β = .34, *∆ R*^*2*^ = .12, *p* = 0.018) to cortisol levels after including quantitative demands to the model.

**Conclusions:**

Person-Environment fit in feminity might be inspected as a predictor for burnout and work engagement. Person-Environment fit in masculinity can be taken into consideration as a predictor for hair cortisol as stress biomarker. Feminity and masculinity can be used as personality traits as well as characteristics of work environment, thus providing a particular gender-role related method of differentiation within gender groups. Also, specific methods could be derived for stress and burnout prevention and promotion of work engagement. Representative population studies with bigger samples and longitudinal surveys are needed to better explore the benefits and limitations of this approach.

**Supplementary Information:**

The online version contains supplementary material available at 10.1186/s12995-021-00303-5.

## Background

In the last decades different phenomena have emphasized the relevance of gender research in relation to stress and burnout. The growing numbers of burnout and psychological illnesses reveal the importance of the related fundamental research [1]. Additionally, the increasing entry of women into the labor market and the ongoing war for talents in the industrial countries [2] reinforce the need for more appropriate approaches to address these health issues by occupational medicine and psychology. To sensitize the public to the issues of sex and gender research the European Research Council hosted a special workshop in November 2020 [3].

Proceeding research shows a tendency for higher stress and burnout levels as well as poorer psychological health scores for women compared to men [4, 5]. In most studies stress coping strategies and health strains have been compared for female and male groups, also considering the gender congruency of the individuals and their work environment, e.g. female employees with male supervisors or women in male-dominated teams and organizations [4]. These studies were based on individuals’ biological sex assuming that individuals of the same sex automatically involve the same cultural and social gender role. With this assumption no differentiation is made within gender groups, thus not considering diversity among women and among men.

Another research direction provides a more specified view on gender-related characteristics since the 1970s, as feminity and masculinity as personality dimensions are focused on, instead of the very rough differentiation of sex or gender groups. Our study follows this scientific argumentation, also picking up the idea of lacking congruence between the individuals and their work environment as a possible stress factor. Accordingly, individual and work environment feminity and masculinity were used to calculate two P-E fit scores analyzed as predictors for subjective health strain measures, hair steroids as biological stress markers, and work engagement.

### Person-environment fit

The construct Person-Environment fit can be generally explained as congruence between the individual and its environment [6]. Theory of Work Adjustment [7, 8] describes it with the term correspondence, other theories refer to it as fit. Most of the person-environment (P-E) fit models focus on the relation between the employee’s needs, wishes, goals on the one side and the benefits, conditions, and supplies a job is offering on the other side. In conclusion, different types of P-E fit refer to various aspects of work environment e.g., person-group fit, person-supervisor fit, or person-organization fit as well as demands-abilities fit, so P-E fit can be described as a multidimensional construct. The observed outcomes in these models are usually job satisfaction [7, 8] and employee stress along with health measures like burnout [9].

Person-Environment fit is usually operationalized by calculating a fit index, subtracting an individual score from the environment score, often additionally transformed by squaring and logarithmising [10] to meet the criteria for linear regression. Accordingly, in this study P-E fit in feminity and P-E fit in masculinity were calculated by squaring and logarithmising the difference of individual and environmental feminity and masculinity levels.

### Gender-role orientation

In the present study, two P-E fit scores ​​are calculated based on the two dimensions of the gender role orientation. Gender-role orientation or gender identity describes the individual self-perception in relation to femininity and masculinity. It reflects how strongly an individual identifies with gender stereotypes and gender roles. The psychosocial aspects culturally associated with feminity and masculinity refer to the bi-polar constructs communion and agency [11, 12]. Communion focuses on relationship-oriented characteristics such as participation, community, forming emotional connections, affirmation of feelings, and is stereotypically related to feminity. Agency follows principles like individualism, a positive picture of oneself, self-assertion, discipline, suppression of feelings, and can be described as task orientation, which refers to masculinity. Other frequently used terms are expressivity (for feminity) and instrumentality (for masculinity) [13], in this article the terms feminity and masculinity are applied consistently. Gender Role Orientation Scale GTS+ applied in this study is a German questionnaire containing socially desired feminine and masculine adjectives [14].

Most studies reveal best mental health [15, 16], highest self-esteem scores [17–19], highest levels of social competence [20] and better adjustment [21] for individuals with both high feminity, and high masculinity. In this context the term androgyny is applied for balanced and high individual levels of feminity and masculinity.

Among subjects with one-sided gender-role orientation, study results show higher health and well-being scores for individuals with high levels of masculinity and low feminity compared to individuals with high feminity and low masculinity. Additionally, masculinity shows higher effects on health measures than feminity and stronger positive relationship with self-esteem and adjustment [15–21].

Previous scientific debate gives various explanations in this matter. Among other reasons, in general masculine characteristics lead to a more positive self-evaluation and higher self-esteem. As Cook describes it “masculinity has a more powerful impact on how positively you see yourself” (p. 477) [19]. This interpretation of relations between masculinity and mental health is called masculine supremacy effect [19] or a “masculine bias” [22].

### Burnout

Job Demands-Resources (JD-R) model assumes that personality dimensions and social processes at work are operating as job resources and demands. According to the JD-R model, job demands mostly affect employees’ burnout while job resources mainly have effects on work engagement [23, 24].

Studies proved burnout as a two-dimensional construct in the model [25–27]. The two dimensions are exhaustion with physical fatigue symptoms, and cynicism, which leads to cynical and negative attitudes towards work. In this study burnout is measured by the validated DearEmployee-Survey questionnaire [28].

Some specific variables related to burnout were identified – among those low support, high job demands and high workload, low autonomy or job control, low reward, and job insecurity [29, 30]. Another job demand related to burnout is high role conflict [31, 32]. Role conflicts appear in job positions with contrary and conflicting work requirements, conflicting goals, and behaviors.

### Work engagement

In JD-R theory work engagement is described with three dimensions. Two burnout-contrary dimensions are vigor and dedication. Vigor is described by a high energy level and readiness to invest in work even in challenging situations. Dedication refers to meaningfulness seen in work, as well as perceived enthusiasm and inspiration. The third work engagement dimension is absorption, meaning a state of high concentration and immersion in work [33].

Research reveals positive and negative impacts of work engagement. Highly engaged employees report positive emotional states connected to their work, and proactively change their work environment to create job resources. They show better performance, have better health, less accidents, and their clients report a higher customer satisfaction [23, 24, 34].

Nevertheless, diverse studies proved that individuals with high work engagement tend to have more overtime-work, which can lead to health strains. In fact, studies reveal a higher work-to-family-conflict for highly engaged employees as well as a raised risk of burnout. In short, there seem to be special conditions on a personal and on the organization level, where work engagement may lead to a negative development [34, 35].

### Steroid levels in hair as stress biomarkers

Another way to determine individuals’ stress reaction is the usage of biological measurements. Assessment of steroid levels in bodily fluids secreted by the activity of the hypothalamus–pituitary–adrenal neuroendocrine axis during the physiological stress response plays an essential role. Human hair has been used for long-term toxicological analyses [36] since the development of applicable methods in the 1980s, due to the known hair’s ability to store substances temporarily present in blood. As human hair grows 1 cm/month (0.39 in) on average [37] hair analysis gives – depending on hair length –information about substance exposure during the last months or years. The most common techniques in substance detection are immunochemical techniques, Gas Chromatography–Mass Spectrometry (GC–MS), and Liquid Chromatography-Mass Spectrometry (LC–MS) [36]. The ongoing further development of these methods also enabled detection of steroids like cortisone, estradiol, testosterone, dehydroepiandrosterone (DHEA), progesterone and others in human and animal hair samples [38–42].

Previous research could prove cortisol levels in hair provide a valid and reliable biomarker of the crucial long-term stress explosion [43, 44]. However, apart from cortisol, study results do not present a consistent picture about correlations between physical, mental, and subjective stress and raised levels of other steroids yet [38–42].

### Effects of P-E fit in feminity and masculinity

Studies regarding gender congruence between individual and work environment focus on the social role differences between men and women based on their biological sex. Consequently, in this research gender-congruency between the individual and supervisor or individual and team refers to men and women working in male or female-dominated working environments, none of them indicating differences in feminity or masculinity. Tough, as higher expressivity scores could be proved for women and higher instrumentality levels for men [14, 45, 46], similar results can be expected for individuals with high P-E fit scores in feminity and masculinity.

Previous research verified that women in male-dominated industries had more stress and poorer mental health scores then male employees in the same job-settings [47]. Other studies showed that subjective stress levels were higher for women in male-dominated working environments [45, 48] as well as elevated sick leave levels among women in extremely male-dominated occupations [49]. A higher sickness absence was also found among men in female-dominated work settings [45, 49]. However, a meta-analysis of 183 studies showed that gender differences do not occur in occupations dominated by the same gender [4].

In conformity with P-E fit theories, a low person-environment fit can lead to an increased adjustment effort resulting in higher levels of perceived stress. Therefore, the higher a P-E fit score (meaning lower fit), the more stress, burnout or other health complaints can be expected.

P-E fit research focuses on subjective stress measures as outcomes and provides no studies on hair steroid levels in this context. Nevertheless, above explanations lead to the conclusion that, a higher P-E fit related to gender-role-orientation leads to higher levels of subjective stress, and this should manifest in physiological stress measures. As hair cortisol is a proven biomarker for long term stress levels [43, 44], higher P-E fit scores in feminity and masculinity (indicating a lower fit) should be related with higher hair cortisol levels. The interdependency with other hair steroids needs to be specified, as previous research provides no consistent picture.

P-E fit related to gender-role-orientation has not been explored regarding work engagement so far. However, some studies investigated other scores describing a more general P-E fit between the individual and its work environment, and the effects of those P-E fit scores on engagement. A general person-organization value congruence describes the level of accordance of individual and organizational values. Value congruence and work engagement were proved to be positively correlated with each other [50]. Person-organization value congruence could be proven as a moderator between engagement and burnout [35].

### Present study goals

**Fig. 1:**
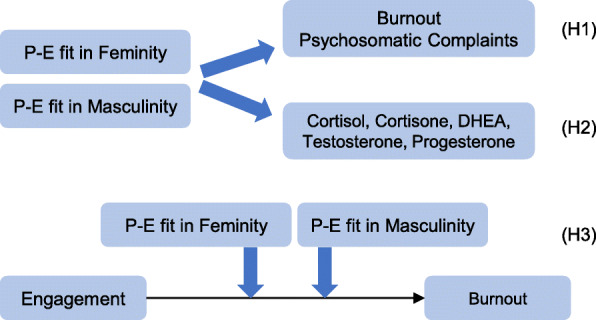
Verified hypotheses on the effects of P-E fit in feminity and masculinity

The observations described above lead to the following hypotheses as shown in Fig. 1. The first hypothesis (H1) claims that P-E fit feminity and masculinity have positive effects on burnout and psychosomatic complaints after considering age and work characteristics. The second hypothesis (H2) declares that P-E fit feminity and masculinity have positive effects on steroid measures in hair after considering age and work characteristics. The third hypothesis (H3) states that P-E fit feminity and masculinity show an effect on work engagement and enhance the negative relation between work engagement and burnout as a moderator variable in a sample of non-managerial employees.

To sum up, P-E fit in femininity and masculinity are used as predictors to analyze their effects on target variables in the study. This is advantageous over the mere gender group recording in stress research because in this way a gender-related diversity within the gender groups can be considered more specifically.

This also might offer an alternative to stress and burnout cause research, since gender groups are not used as predictors but gender-role-related characteristics of individuals and the work environment. A distinction of the stress and burnout values ​​among gender groups only allows a descriptive comparison. By identifying effects of P-E fit in femininity and masculinity, appropriate and more specific approaches in stress and burnout prevention and promotion of work engagement can be developed.

Another special feature of the study is that reference is made to subjective stress values ​​(e.g. burnout) as well as hair steroids ​​as stress biomarkers.

## Methods

### Study design, recruitment of participants

To prove the hypotheses a cross-sectional study was implemented and has been described in detail in an earlier publication [51], a brief method outline is given in this article.

A survey was carried out in a German medical services company addressing all 411 employees. A total of 171 workers answered the online query (42%). The sample involves 146 women who answered a self-assessment questionnaire, 58 of them (40%) submitted hair samples for steroid detection. The participation was voluntary, no agreements were made with participants, no payments were made to them. All female participants answering the online query or submitting hair samples were considered part of the study, no restrictions leading to exclusion—except biological sex—were made.

### Measures

#### Predictors

P-E fit feminity and masculinity were investigated as predictors in all three hypotheses. GTS+ scale [14] was used for measuring feminine and masculine gender role orientation. First, the participants were asked to describe themselves answering the questionnaire to measure individual feminity (α = .82) and individual masculinity (α = .83). In a second step, the subjects characterized their work environment including colleagues and supervisors using the same scale. Thus, environmental feminity (α = .85) and environmental masculinity (α = .86) were measured. To operationalize P-E fit score, the individual rating was subtracted from the subjective rating for the work environment. In a next step, the values were transformed by squaring and logarithmising to meet the requirement of a linear relationship for regression analysis. This way, the two predictor variables P-E fit in feminity and P-E fit in masculinity were calculated. Both variables do not differentiate between cases with higher scores in individual or environmental feminity and masculinity. The predictors rather quantify a general fit. This way, high values in both P-E fit scores represent a bigger difference between the individual and environment feminity or masculinity. Accordingly, low values indicate less difference and a better fit.

Considered work characteristics include the following variables: Activity scope, task completeness, development chances, workplace ergonomics, working time flexibility, work interruptions, work material supply, information supply, versatility of cognitive tasks, role clarity, physical exhaustion, work-qualification equivalency, quantitative work demands, role conflicts, work organization, task variety, work-life balance, and work environment strains (for more detailed information, see Table 1 and Table 2, Additional file 1). All work characteristics were quantified by the newly validated DearEmployee-Survey questionnaire, which was developed to identify occupational demands and consists of dimensions which are related to psychological strain in the workplace as well as work engagement [28]. Among these work characteristics five variables revealed internal consistency of Cronbach’s Alpha lower than 0.70, indicating an inter-relatedness, which is possibly below the satisfactory level [52]: task completeness, role clarity, quantitative work demands, task variety, work organization (see Table 1, Additional file 1). Accordingly, these variables were removed leaving 13 work characteristics considered in the analysis.

**Table 1:** Pearson’s correlations of P-E fit in feminity and masculinity, health strains, hair steroids and age

Variable	1	2	3	4	5	6	7	8	9	10	11
1 FFem^a^	1										
2 FMasc^b^	.01	1									
3 Burnout	.31***	.02	1								
4 Complaints	.12	−.05	.44***	1							
5 Cortisol	.28	.32*	<.01	−.08	1						
6 Cortisone	.16	.20	.12	.24	.73***	1					
7 DHEA	−.02	−.08	−.02	−.05	−.07	−.29*	1				
8 Testosterone	−.18	−.12	−.40	.31	.69	.29	.63	1			
9 Progesterone	−.23	.03	−.04	−.13	−.08	−.23	.26	.36	1		
10 Engagement	−.25**	−.05	−.44***	−.37**	.03	.12	−.23	−.15	−.24	1	
11 Age	.31***	−.23**	.32***	.18	.12	.15	−.07	.09	−.25	−.01	1

^a^ P-E fit in feminity ^b^ P-E fit in masculinity; *p < .05, ** *p <* .01, *** *p <* .001

**Table 2:** Hierarchical linear regression models predicting burnout

Predictor	Model 1a		Model 1b	
	∆*R*^*2*^	β^1^	∆*R*^*2*^	β^1^
Step 1	.10***		.09***	
Age		.20*		.02**
Step 2	.33***	.	.33***	
Activity scope		< .01		.01
Development chances		< .01		< .01
Workplace ergonomics		−.01		−.01
Working time flexibility		< .01		−.01
Interruptions		.02		.02
Material supply		.01		.01
Versatility of cognitive tasks		< .01		< .01
Information supply		.01		.01
Physical exhaustion		.01		.02*
Work-qualification equivalency		.01		.01
Role conflicts		.02*		.03***
Work-life balance		.02*		.01
Work environment strains		.02		.01
Step 3	.04**		< .01	
P-E fit in feminity		.22**	–	–
P-E fit in masculinity	–	–		< .01
Total *R*^*2*^	.46***		.40***	
*n*	122		125	

*Note*. * *p* < .05, ** *p* < .01, *** *p* < .001. ^1^ β in the model including all steps

#### Outcomes

In the study burnout and number psychosomatic complaints (H1), hair steroids including cortisol, cortisone, dehydroepiandrosterone [DHEA], testosterone as well as progesterone (H2), and work engagement (H3) are analyzed as outcomes (see Table 1 and Table 2, Additional file 1 for detailed information).

To identify burnout, psychosomatic complaints, and work engagement the DearEmployee-Survey [28] was used in the study. Specific psychosomatic complaints were addressed in the questionnaire (headache, neck or back discomfort, gastrointestinal complaints, cardiovascular complaints, dizziness, restlessness, fear, panic, tight feeling in the throat, sleep problems, nightmares, concentration problems, strong weight changes, facial muscle twitching). In the study, the number of psychosomatic complaints specified in the answers serves as an outcome variable in H1. To measure steroids, hair samples were taken and analyzed by Liquid Chromatography–Mass Spectrometry (LC-MC/MS) [36, 44] in a specialized laboratory. A more specific method description is given in a previous article [51].

### Statistical methods

To prove H1 and H3, collected data (see Additional file 2) was analyzed using hierarchical linear regression models. A sample size of 139 subjects was calculated a priori for linear regression models (α = 0.05, power = 0.80, *R*^2^ = 0.13) with 15 predictors (P-E fit score, age, 13 work characteristics), and a sample size of 55 subjects for a simple linear regression. For the models of hierarchical regression, predictors were added to the model step by step. This approach verified whether P-E fit feminity and masculinity still added considerable variance explanation to the outcomes after considering persons’ age as control variable (1st step) and 13 work characteristics (2nd step). As target parameter the added variance explanation (∆ *R*^*2*^) compared to the previous model was proven for significance. P-E fit feminity and masculinity were added to the models in the last step.

Assumptions underlying these statistical procedures such as linear relationship of the variables, normal distribution of residuals, homoscedasticity, absence of highly influential values and multicollinearity were tested.

To prove H3 (see Fig. 1) –based on previous research–P-E fit in feminity and masculinity were proven as moderators between burnout and work engagement. Variables were centered to avoid multicollinearity issues in moderator analyses and to optimize the interpretation of standardized estimates. First, a linear regression model with P-E fit in feminity/masculinity as moderator and work engagement as predictor for burnout was calculated. In a second step, an interaction term of moderator and predictor was included in the model. The added variance explanation (∆ *R*^*2*^) compared to the previous model was proven for significance as target parameter [53].

Statistical software R Studio [54] using packages R Commander [55–57], and pwr [58] were applied for data analyses.

## Results

The 146 female participants were 22 to 66 years old (*M* = 40.48, *SD* = 10.38, *R =* 44), six participants did not mention their year of birth (4%). Male participants (19 subjects, two of them provided hair samples) and 6 participants not identifying their gender were excluded from the analysis (15% of 171 subjects taking part in the query).

Nearly all subjects–136 out of 146–had a permanent working contract in the company (93%), three participating employees had a fixed-term working contract (2%), three other participants had a working contract allowing a maximum wage of 400 Euros (2%), one person was working on probation (< 1%), two of the query participants were student assistants (1%). Above all female subjects 8 participants (6%) confirmed having a side income.

The sample of 58 female participants delivering hair samples is described in the following. In this group two subjects age was “less than 24 years” (3%), 12 subjects were “25–34 years” old (21%), 21 subjects were “35–44 years” old (36%), the age of 15 subjects was “45–54 years” (26%), and eight subjects were “55–64 years” old (14%). The marital status of 29 participants was “single “(50%), 21 subjects were „married “(36%), three subjects stated to be „married, but living separated “(5%), and five participants indicated themselves as „divorced “(9%).

The study focuses on the effects of P-E fit in feminity and P-E fit in masculinity on burnout, psychosomatic complaints, hair steroids, and work engagement. Descriptive statistics are stated in Table 1 and Table 2, Additional File 1. An overview of Pearson’s bivariate correlations between predictors, burnout, psychosomatic complaints, hair steroids, work engagement, and age as a control variable is given in Table 1. P-E fit in feminity correlates significantly with burnout as well as age and has a negative correlation with work engagement. P-E fit in masculinity correlates significantly with cortisol and age (negative interrelation). DHEA, testosterone and progesterone have no significant relations with any of the predictor variables.

Table 3 in Additional File 1 shows correlations between P-E fit in feminity and masculinity, burnout, complaints, work engagement, age, and work characteristics. P-E fit in feminity shows poor correlations with development chances, workplace ergonomics, working time flexibility, and information supply. No significant correlations could be found between any work characteristics variables and P-E fit in masculinity. Intercorrelations between job demand variables are summarized in Additional File 1, Table 4.

Hierarchical linear regression models (see Table 2 and Table 3) were inspected to analyze the variance explanation of P-E fit in feminity and masculinity (after considering age and work characteristics) for the outcome variables.

The first hypotheses (H1) claims predictor effects on subjective health strain measures burnout and complaints, age and work characteristics were included in the analysis. A strong variance explanation could be verified for burnout in a hierarchical multiple regression model - Table 2 provides an overview. After age and work characteristics, P-E fit in feminity still added significant variance explanation on burnout (Model 1a, Table 2). P-E fit in masculinity could not be proven as a significant predictor for burnout (Model 1b, Table 2). Regarding psychosomatic complaints, no significant variance explanation could be proven either for P-E fit in feminity (*R*^*2*^ = .01, *p* = .195) nor P-E fit in masculinity (*R*^*2*^ < .01, *p* = .596).

The second hypothesis (H2) claims predictor effects on hair steroids including cortisol, cortisone, DHEA, testosterone and progesterone. A hierarchical linear regression model calculation explaining the variance in hair cortisol was abandoned as no sufficient power could be promoted due to the 58 provided hair samples*.* However, this sample size enabled a simple linear regression calculation. P-E fit in feminity explained no significant variance of any hair steroid levels: cortisol (*R*^*2*^ = .08, *p* = .079), cortisone (*R*^*2*^ = .03, *p* = .291), DHEA (*R*^*2*^ = .03, *p* = .276), testosterone (*R*^*2*^ = .01, *p* = .418) and progesterone (*R*^*2*^ = .06, *p* = .098)*.*

Significant variance explanation of cortisol was provided by P-E fit in masculinity (β = .33, *R*^*2*^ = .10, *F* (1, 45) = 5.24, *p =* .027, 95% CI [0.17, 2.73]). No significant effects P-E fit in masculinity could be verified on other steroid levels: cortisone (*R*^*2*^ = .04, *p* = .155), DHEA (*R*^*2*^ < .01, *p* = .640), testosterone (*R*^*2*^ = .05, *p* = .126) and progesterone (*R*^*2*^ < .01, *p* = .939).

The third hypothesis (H3) states predictors’ variance explanation on work engagement as well as predictors’ moderator effects on the relation between work engagement and burnout.

We could attest poor variance explanation of work engagement by P-E fit in feminity *(*β = −.29, *R*^*2*^ = .09, *F* (1, 136) = 12.85, *p <* .001, 95% CI [−1.50, −0.28]), and no significant variance explanation by P-E fit in masculinity (*R*^*2*^ < .01, *p* = .551)*.* In multiple regression models involving age and work characteristics neither P-E fit in feminity nor P-E fit in masculinity revealed statistical relevance (see Table 5, Additional file 1). The analysis did not approve any moderator effects (see Table 6 and Table 7, Additional file 1).

## Discussion

The first hypothesis, claiming that P-E fit in feminity and masculinity have effects on subjective health strain measures, was only proved for P-E fit in feminity having a significant positive effect on burnout. Results showed that P-E fit in feminity still added significant variance explanation on burnout after considering age and work characteristics. However, the second hypothesis, declaring that P-E fit in feminity and masculinity have positive effects on hair steroids as biological stress markers, could only be verified for P-E fit in masculinity having a significant positive effect on hair cortisol. Study results partly supported the third hypothesis claiming that P-E fit in feminity predicts work engagement. However, no moderator effects could be verified. Similarly, no effects of P-E fit in masculinity could be proved on work engagement.

As P-E fit in feminity was proved adding significant variance explanation to burnout, our research could provide an advanced and more specified explanation for usually identified higher burnout levels in woman [4, 5]. For, if female employees have higher feminity scores than their male colleagues [14, 45, 46], in a work environment with perceived low feminity, this could result in elevated P-E fit in feminity (a bigger difference in individual and environment score) in the group of women. Thus P-E fit in feminity – and not female gender – possibly manifests in higher burnout levels in women. Further, our study might show a new way to explore reasons for burnout in general – e.g., also for male employees with high feminity levels in work environments with low feminity.

Our study results cannot directly explain commonly reported higher cortisol levels in men compared to women [59] as P-E fit in masculinity showed positive effects on cortisol levels. Male individuals usually have higher masculinity levels compared to women [45, 48]. In a work environment with perceived high masculinity this would lead to lower P-E fit levels (less difference in masculinity). As the study involved a female sample, a possible conclusion could be drawn that lower cortisol levels are usually reported for women compared to men as women might tendentially not perceive a big difference in their individual and work environment masculinity. Thus, it would possibly rather be more reasonable to raise female characteristics in company and team culture then to change masculine characteristics to prevent burnout in women.

Using P-E fit in feminity and masculinity instead of gender congruence in research has several benefits. Feminity as a characteristic for focusing on a connection to individual emotions and emotions in others, and masculinity as a dimension for focusing on a positive picture of oneself, on the individual goals may be viewed as attributes of company culture, as individual and group competencies.

One practical implication for occupational health medicine and psychology as well as human resources development is to consider a close intercommunion between the two areas. An example could be taken from the diversity management approach in which economic decisions can be made considering health aspects and vice versa [60, 61]. Further practical implications can be derived for working culture, team composition and team development for a healthier work environment. Improved and specific approaches to lower occupational stress and burnout levels could contribute to the prevention of psychological illnesses, which are an ongoing public health issue in the last decades. Not to forget, it might be beneficial to raise feminity characteristics in the work environment and company culture in order to increase work engagement.

To raise feminine characteristics in company culture requires actions communicating an interest in employees’ emotions and wellbeing, apart from work duties and goals. As an example, team building events can promote such connections between colleagues as well as between managers and staff members. However, a communicated interest for employees’ wellbeing apart from job tasks is expressed on daily basis. It is based on leadership style, social competencies of the managers and team members as well as connected to the company culture in general. Accordingly, specific recommendations on these competencies could be developed to increase employee work engagement, which relates positively with lower burnout levels and better performance. In addition to this, companies also benefit from this knowledge by improving their work environment, thus positioning themselves as a desirable employer to address the shortage of skilled employees.

Apart from that, the indicated effects of job characteristics on burnout and hair steroids as stress biomarkers are not surprising. The results expose already known burnout correlated work characteristics, such as age and role conflicts (see Table 2) as frequently shown by burnout research [29, 30, 62]. As in previous research [43], we could prove effects on cortisol as a biomarker for stress, but not on other steroids. There is a wide range of studies investigating the effects of subjective stress measures on cortisone, DHEA, testosterone, and progesterone [38–42] with contradictory findings. However, no previous studies analyzed P-E fit in feminity and masculinity as predictors for hair steroids.

Our study confirmed P-E fit in feminity explaining significant variance in work engagement. This corresponds to other study results involving general person-organization value congruence [50]. Employees’ P-E fit in feminity might play a special role in connection with work engagement, as P-E fit in masculinity did not show a similar effect.

### Study strengths and limitations

It must be considered that only 58 subjects provided hair samples in the study. Sufficient power of 80% could not be always ensured in the statistical analyses regarding hair steroid levels, since Pearson’s correlation and linear regression models with more than one predictor require a bigger sample size. This way, some existing correlations and regression effects – especially those regarding effects on hair steroids (H2) – may not be discovered.

As the moderator effects of P-E fit in feminity and masculinity on work engagement were not supported, we need to consider that previous studies show correspondent moderator effects for general person-organization value congruence [35]. This may not be applicable for the specific P-E fit measures used in our study.

Another critical point is the calculation of P-E fit in feminity and masculinity, where squaring and logarithmising were involved. This transformation was necessary to meet the requirements for linear regression. However, after these steps no difference can be made in cases where one’s own gender role orientation level is rated higher than gender role orientation of the job environment or vice versa. This needs to be noted critically, as a deficit could lead to a different attitude as an outcome than rather than the same amount of an oversupply. This could be crucial to investigate the interdependencies of the variables.

Further, the cross-sectional observational study design only allows conclusions about interrelations of the variables, no causal effects (e.g., as shown in the regression models) can be verified.

For result generalization, it is particularly important that the chosen sample were women employees in a medical services company. As psychological, social, and biological aspects of biological sex and gender are crucial in this research as well as specific job characteristics and company culture, a broad generalization of the study results is not appropriate.

To emphasize the advantages of the study, not only relations between individual feminity, masculinity and mental health were analyzed, as it is the case in previous research. Gender-role associated work environment and individual characteristics were both linked to each other in calculating P-E fit in feminity and masculinity, and analyzed as a predictor for stress, burnout, and work engagement.

Additionally, the study might improve occupational stress research in relation to gender congruence studies, since previous studies relate to biological sex and social role of male and female individuals as stress predictors, not differentiating within gender groups. Whereas our study focuses on effects of P-E fit in gender-role orientation, which could show a more sophisticated way to identify actual individual and work environment characteristics predicting stress and burnout.

Research of sex or gender congruence in occupational environment as predictors for stress and health strains only enables a descriptive comparison. The only practical implication, which can be derived from gender congruence research is a broader inclusion and diversity in different business areas. While this is a reasonable step, this will probably not be the final solution as relevant differentiation is needed within gender groups as well as in the description of company culture. To put it simply, more specific tools are needed to deal with the relevant present-day challenges in occupational stress prevention.

### Future research

To gain a more comprehensive picture, in the future it might be reasonable to investigate the effects of P-E fit in feminity and masculinity on hair steroids with bigger samples. Especially cortisol should be focused on, but also interrelations between P-E fit, burnout, and other hair steroids could be specified in further studies.

Apart from the sample size, sample characteristics – such as biological sex, gender, age – should also be adapted to bigger target populations to enable a broader result generalization. It might be especially interesting, if male subjects with a high P-E fit in feminity show similar effects as female subjects.

Further research could also reveal, if the discussed effects can be differentiated depending on if feminity or masculinity are rated higher for individual or job environment, thus giving fit scores a more specific differentiation. Longitudinal observations should be undertaken to further investigate long-term effects.

In summary, the study shows effects of P-E fit in feminity on burnout and work engagement as well as effects of P-E fit in masculinity on biological stress markers. Further research with larger and more diverse samples as well as longitudinal studies are needed to explore the advantages and the limits in this approach. P-E fit in feminity and P-E fit in feminity could represent improved methods in gender-related stress research. Both scores might be considered as relevant predictors, which could improve stress and burnout prevention and promote work engagement as specific interventions could be derived.

## Conclusions

The presented study leads to the conclusions that on one hand P-E fit score in feminity possibly represents a considerable predictor variable for burnout and work engagement. On the other hand, P-E fit scores in masculinity could be considered an important predictor variable for cortisol measures in hair as biological stress markers.

## Supplementary Information

**Additional file 1: Table S1***.* Descriptive statistics of predictors, work characteristics and age. **Table S2***.* Descriptive statistics of health strains, hair steroids, and job engagement. **Table** ***S*3***.* Pearson’s correlations with work characteristics. **Table S4**. Intercorrelations of work characteristics. **Table S5**. Regression models for work engagement. **Table S6.** P-E fit in Feminity as Moderator. **Table S7.** P-E fit in Masculinity as Moderator.

**Additional file 2:.** Data collected and analyzed in the study.

## Data Availability

Data generated and analyzed during this study are included in this published article and its supplementary information, Additional file 2.
