# Supplementary material for: Effects of person-environment fit of gender-role orientation on burnout, engagement and hair steroids as stress biomarkers among women
Source: J Occup Med Toxicol. 2021 Apr 16;16:13. doi: 10.1186/s12995-021-00303-5 (PMC8050924; doi:10.1186/s12995-021-00303-5)
Supplement: Supplementary file 1 — Additional file 1: Table S1. Descriptive statistics of predictors, work characteristics and age. Table S2. Descriptive statistics of health strains, hair steroids, and job engagement. Table S3. Pearson’s correlations with work characteristics. Table S4. Intercorrelations of work characteristics. Table S5. Regression models for work engagement. Table S6. P-E fit in Feminity as Moderator. Table S7. P-E fit in Masculinity as Moderator. [file 12995_2021_303_MOESM1_ESM.docx]

**Table 1 Descriptive statistics of predictors, work characteristics and age.**

| Variable | n | Missing values^1^ | M (SD) | 95% CI | Range | Cronbach's α |
| --- | --- | --- | --- | --- | --- | --- |
| Predictors | | | | | | |
| P-E fit in feminity | 143 | 3 | 0.55 (0.51) | [0.46, 0.63] | 2.38 | - |
| P-E fit in masculinity | 142 | 4 | 0.58 (0.48) | [0.50, 0.66] | 2.43 | - |
| Work characteristics | | | | | | |
| Activity scope | 146 | 0 | 6.93 (2.49) | [6.52, 7.33] | 10.00 | .84 |
| Task completeness | 146 | 0 | 7.21 (2.06) | [6.88, 7.54] | 10.00 | .35 |
| Development chances | 145 | 1 | 6.66 (2.62) | [6.23, 7.09] | 10.00 | .86 |
| Workplace ergonomics | 146 | 0 | 6.46 (2.41) | [6.07, 6.85] | 10.00 | .78 |
| Working time flexibility | 146 | 0 | 6.71 (2.69) | [6.28, 7.15] | 10.00 | .77 |
| Interruptions | 146 | 0 | 6.08 (2.70) | [5.64, 6.52] | 10.00 | .86 |
| Material supply | 146 | 0 | 8.31 (2.09) | [7.98, 8.65] | 9.00 | .84 |
| Versatility of cognitive tasks | 146 | 0 | 8.14 (2.29) | [7.77, 8.52] | 9.00 | .86 |
| Information supply | 146 | 0 | 3.93 (2.43) | [3.54, 4.32] | 9.33 | .89 |
| Role clarity | 146 | 0 | 2.67 (1.70) | [2.40, 2.94] | 7.67 | .57 |
| Physical exhaustion | 146 | 0 | 5.15 (2.35) | [4.76, 5.53] | 10.00 | .78 |
| Work-qualification equivalency | 144 | 2 | 8.19 (2.37) | [7.80, 8.57] | 10.00 | .81 |
| Quantitative work demands | 146 | 0 | 6.68 (2.16) | [6.34, 7.02] | 10.00 | .69 |
| Role conflicts | 146 | 0 | 3.51 (2.22) | [3.15, 3.87] | 8.33 | .81 |
| Task variety | 146 | 0 | 6.21 (2.03) | [5.88, 6.54] | 10.00 | .66 |
| Work-life balance | 146 | 0 | 2.73 (2.42) | [2.33, 3.12] | 10.00 | .84 |
| Environment strains | 146 | 0 | 5.23 (2.46) | [4.83, 5.63] | 10.00 | .82 |
| Work organization | 145 | 1 | 7.10 (1.91) | [6.79, 7.41] | 9.33 | .66 |
| Control variable | | | | | | |
| Age | 140 | 6 | 40.48 (10.38) | [38.76, 42.20] | 44 | - |

Note. CI = confidence interval. ^1^All missing data can be considered as missing completely at random.

**Table 2 Descriptive statistics of health strains, hair steroids, and job engagement.**

| Variable | n | Missing Values^a^ | M (SD) | 95% CI | Range | Cronbach's α |
| --- | --- | --- | --- | --- | --- | --- |
| Health strains | | | | | | |
| Burnout | 144 | 2 | 4.96 (2.49) | [4.55, 5.36] | 10.00 | .90 |
| Number of complaints | 145 | 1 | 2.35 (1.36) | [2.13, 2.57] | 6.00 | - |
| Hair steroids | | | | | | |
| Cortisol | 58 | 0 | 4.77 (5.94) | [3.24, 6.30] | 36.99 | - |
| Cortisone | 58 | 0 | 11.59 (10.95) | [8.77, 14.40] | 72.47 | - |
| DHEA | 58 | 0 | 10.42 (7.90) | [8.39, 12.45] | 36.86 | - |
| Progesterone | 58 | 0 | 2.58 (3.48) | [1.69, 3.48] | 21.47 | - |
| Testosterone | 58 | 0 | 0.05 (0.12) | [0.02, 0.08] | 0.62 | - |
| Work engagement | | | | | | |
| Engagement | 143 | 3 | 8.74 (1.40) | [8.51, 8.97] | 7.20 | .77 |

Note. CI = confidence interval. ^a^All missing data can be considered as missing completely at random.

**Table 3 Pearson’s correlations with work characteristics.**

| Work characteristics | Predictors^a^ | |  | Outcomes^b^ | | | | | | | | | |  | Control  variable |
| --- | --- | --- | --- | --- | --- | --- | --- | --- | --- | --- | --- | --- | --- | --- | --- |
|  |  |  |  | H1 | |  | H2 | | | | |  | H3 |  | Age |
|  | 1 | 2 |  | 3 | 4 |  | 5 | 6 | 7 | 8 | 9 |  | 10 |  |  |
| Activity scope | -.07 | < .01 |  | .02 | -.14 |  | .04 | -.03 | .07 | -.02 | .02 |  | .31* |  | < .01 |
| Development chances | -.25** | .10 |  | -.15 | -.15 |  | .02 | -.02 | -.14 | .09 | .23 |  | .42*** |  | -.14 |
| Workplace ergonomics | -.18* | .04 |  | -.22** | -.07 |  | .27 | .23 | -.14 | .27* | .26* |  | .31** |  | -.04 |
| Working time flexibility | -.18* | .03 |  | -.03 | .07 |  | -.12 | .02 | -.12 | -.01 | -.17 |  | .26** |  | .02 |
| Interruptions | .09 | .13 |  | .43*** | .26** |  | .07 | -.07 | .18 | -.02 | -.09 |  | -.27** |  | .22** |
| Material supply | .01 | .02 |  | -.13 | -.07 |  | -.04 | -.28* | -.02 | .13 | .29* |  | .26** |  | -.06 |
| Versatility of cognitive tasks | -.03 | -.05 |  | .16 | .19* |  | .17 | .15 | -.20 | -.12 | .00 |  | .30*** |  | .18* |
| Information supply | .21* | -.06 |  | .36*** | .13 |  | -.14 | .01 | -.05 | -.03 | -.16 |  | -.51*** |  | .21* |
| Physical exhaustion | < .01 | -.16 |  | .25** | .13 |  | .09 | .13 | -.23 | -.09 | -.19 |  | < .01 |  | .03 |
| Work-qualification equivalency | .08 | .02 |  | .03 | -.07 |  | .31* | .25 | -.14 | -.10 | .05 |  | .30*** |  | .05 |
| Role conflicts | .14 | .04 |  | .50*** | .31*** |  | .07 | .08 | .04 | -.23 | -.21 |  | -.34*** |  | .06 |
| Work-life balance | .02 | .04 |  | .07 | -.07 |  | .40* | .33* | -.11 | -.08 | .00 |  | -.20 |  | -.25* |
| Work environment strains | .08 | .06 |  | .22** | .05 |  | .12 | .17 | .01 | -.26 | -.18 |  | -.31*** |  | -.05 |

^a^1: P-E fit in feminity, 2: P-E fit in masculinity.

^b^3: Burnout, 4: Complaints, 5: Cortisol, 6: Cortisone, 7: DHEA, 8: Testosterone, 9: Progesterone, 10: Engagement.

**p <* .05, ** *p <* .01, *** *p <* .001

**Table 4 Intercorrelations of work characteristics.**

| Variable | 1 | 2 | 3 | 4 | 5 | 6 | 7 | 8 | 9 | 10 | 11 | 12 |
| --- | --- | --- | --- | --- | --- | --- | --- | --- | --- | --- | --- | --- |
| 1 Activity scope | 1 |  |  |  |  |  |  |  |  |  |  |  |
| 2 Development chances | .30*** | 1 |  |  |  |  |  |  |  |  |  |  |
| 3 Workplace Ergonomics | .10 | .19* | 1 |  |  |  |  |  |  |  |  |  |
| 4 Working time flexibility | .48*** | .24** | .27*** | 1 |  |  |  |  |  |  |  |  |
| 5 Interruptions | .03 | -.20* | -.22** | -.03 | 1 |  |  |  |  |  |  |  |
| 6 Material supply | .08 | .18* | .40*** | .14 | -.18* | 1 |  |  |  |  |  |  |
| 7 Versatility of cognitive tasks | .37*** | .31*** | .06 | .27*** | .20* | .06 | 1 |  |  |  |  |  |
| 8 Information supply | -.04 | -.37*** | -.30*** | -.14 | .34*** | -.39*** | < .01 | 1 |  |  |  |  |
| 9 Physical exhaustion | -.03 | -.02 | -.05 | -.10 | .04 | .01 | .20* | .17* | 1 |  |  |  |
| 10 Work-qualification equivalency | .19* | .42*** | .04 | .12 | -.08 | .04 | .37*** | -.19* | .08 | 1 |  |  |
| 11 Role conflicts | .06 | -.09 | -14 | .10 | .44*** | -.17* | .10 | .42*** | .16* | -.06 | 1 |  |
| 12 Work-life balance | -.01 | -.09 | -.03 | -.10 | -.05 | -.01 | -.07 | .18 | .06 | -.12 | .12 | 1 |
| 13 Environment strains | -.19* | -.29*** | -.47*** | -.34*** | .21* | -.39*** | -.20* | .21* | .13 | -.05 | .13 | .15 |

*Note.* **p <* .05, ** *p <* .01, *** *p <* .001.

**Table 5 Regression models for work engagement.**

|  | Model 3a including  P-E fit in feminity | Model 3b  including  P-E fit in masculinity |
| --- | --- | --- |
| Predictor | β | β |
| Control Variable Age | .13 | .01 |
| Job Demands | |  |
| Activity scope | .17 | .02 |
| Development chances | -.13 | -.01 |
| Workplace ergonomics | .02* | .02* |
| Working time flexibility | .01 | .01 |
| Interruptions | -.01 | < .01 |
| Material supply | < .01 | -.01 |
| Versatility of cognitive tasks | .16 | .02 |
| Information supply | -.38** | -.04** |
| Physical exhaustion | .01 | .01 |
| Work-qualification equivalency | .02 | .02 |
| Role conflicts | < .01 | < .01 |
| Work-life balance | < .01 | < .01 |
| Work environment strains | -.01 | -.02 |
| Predictors | | |
| P-E fit in feminity | -.01 | - |
| P-E fit in masculinity | - | < .01 |
| Total *R²* | .63*** | .62*** |
| *n* | 88 | 83 |

*Note*. * p < .05, ** p < .01, *** p < .001.

**Table 6 P-E fit in feminity as moderator between engagement and burnout.**

|  | Model 3c  with interaction term | | Model 3d  without interaction term | |
| --- | --- | --- | --- | --- |
| Predictors for Burnout | $\Delta$*R²* | β | $\Delta$*R²* | β |
| Step 1 | .29*** |  | .29*** |  |
| Work engagement  P-E fit in feminity |  | -.45***  .23 |  | -.45***  .21** |
| Step 2 | < .01 |  | - |  |
| Interaction term: engagement  and P-E fit in feminity |  | < .01 |  |  |
| Total *R²* | .29*** |  | .29*** | |
| *n* | 127 |  | 127 | |

*Note*. * p < .05, ** p < .01, *** p < .001.

**Table 7 P-E fit in masculinity as moderator between engagement and burnout.**

|  | Model 3e  with interaction term | | Model 3f  without interaction term | |
| --- | --- | --- | --- | --- |
| Predictor for Burnout | $\Delta$*R²* | β | $\Delta$*R²* | β |
| Step 1 | .29*** |  | .08** |  |
| Work engagement  P-E fit in masculinity |  | -.48***  < .01 |  | -.48***  < .01 |
| Step 2 | .01 |  | - |  |
| Interaction term: engagement and P-E fit in masculinity |  | < .01 |  |  |
| Total *R²* | .24*** |  | .23*** | |
| *n* | 131 |  | 131 | |

*Note*. * p < .05, ** p < .01, *** p < .001.
